# Modeling of the Binding of Peptide Blockers to Voltage-Gated Potassium Channels: Approaches and Evidence

**Published:** 2016

**Authors:** V. N. Novoseletsky, A. D. Volyntseva, K. V. Shaitan, M. P. Kirpichnikov, A. V. Feofanov

**Affiliations:** M.V.Lomonosov Moscow State University, Faculty of Biology, Leninskie Gory 1, bldg. 12, 119992 , Moscow, Russia; Shemyakin-Ovchinnikov Institute of Bioorganic Chemistry, Russian Academy of Sciences, Miklukho- Maklaya str. 16/10, 117997, Moscow, Russia

**Keywords:** blockers, potassium channels, molecular modeling, complex structure

## Abstract

Modeling of the structure of voltage-gated potassium (KV) channels bound to peptide blockers aims to identify the key amino acid residues dictating affinity and provide insights into the toxin-channel interface. Computational approaches open up possibilities for *in silico *rational design of selective blockers, new molecular tools to study the cellular distribution and functional roles of potassium channels. It is anticipated that optimized blockers will advance the development of drugs that reduce over activation of potassium channels and attenuate the associated malfunction. Starting with an overview of the recent advances in computational simulation strategies to predict the bound state orientations of peptide pore blockers relative to KV-channels, we go on to review algorithms for the analysis of intermolecular interactions, and then take a look at the results of their application.

## INTRODUCTION

Potassium (K^+^) channels are pore-forming transmembrane proteins known to mediate cell functions via selectively allowing potassium fluxes across the cell membrane. Potassium channels are ubiquitously expressed in all cell types, contributing to the maintenance of the resting membrane potential, the regulation of cardiac and nerve excitability, the release of neurotransmitters, the contraction of muscles, and the secretion of hormones [1, 2]. Potassium channels play an important role in the diagnosis and treatment of different pathologies [3-6].

The group of human potassium channels includes Ca^2+^-activated channels (K_Ca_), inwardly rectifying channels (K_IR_), and two-pore domain (K_2p_) and voltagegated (K_V_) channels. The latter form the largest family that comprises 12 subfamilies, such as Shaker/K_V_1 (KCNA), Shab/K_V_2 (KCNB), Shaw/K_V_3 (KCNC), etc. They share structural similarities (except K_V_4–K_V_9) and exist as homotetrameric proteins with a four-fold axis of symmetry. The mechanisms by which K_V_-channels are activated have been reviewed in reference [7].

Dysfunctional K_V_-channel activity is implicated in the etiology of a number of human diseases. The pharmacological targeting of ion channels offers abundant opportunity for treatment. For example, these disorders have negative consequences on membrane excitability, as well as cardiac and nerve function [8]. Episodic ataxia type 1, an autosomal dominant neurological disorder, is caused by a mutation of the KCNA1 gene encoding the voltage-gated K^+^ channel K_V_1.1, which substitutes valine for leucine at position 408 [9]. Genetic studies have also identified mutations in KCNQ2 and KCNQ3 encoding the voltage-gated K^+^ channels K_V_7.2 and K_v_7.3, which lead to benign familial neonatal seizures [10]. Gene expression profiles across various stages of Alzheimer’s disease progression showed that K_V_3.4 overexpression (KCNC4) and K_V_3.1 dysfunction (KCNC1) alter the ion currents in neurons and, consequently, synaptic activity, resulting in neurodegenerative sequelae [11]. Voltage-gated K^+^ channel defects (K_V_7.1 (KCNQ1), K_V_11.1 (KCNH2), KCNE1, KCNE2) have been associated with the long Q-T interval syndrome [12]. The Brugada syndrome is a genetic heart disorder resulting from mutations in the KCND3 gene encoding the K_V_4.3 channel [13]. Studies with patients suffering from acute coronary insufficiency identified mutations in the KV1.3 encoded gene [14]. K_V_1.3 inhibitors suppress the proliferation of T-lymphocytes (particularly, effector memory T cells), relieving symptoms of multiple sclerosis, type 1 diabetes, rheumatoid arthritis, psoriasis, and bronchial asthma [15]. The K_V_2.1 channel was therapeutically targeted to mitigate type 2 diabetes [16]. Various snake, scorpion, spider, cone snail, and sea anemone toxins can readily modulate channel gating. These peptide toxins are classified in terms of their mechanism of action: (i) pore blockers binding to and plugging the external mouth of the channel (scorpion and sea anemone toxins); (ii) – peptides interacting with the VSD-domain, locking the channel in the resting state (tarantula venom peptides).

Until recently, only a few crystal structures of K_V_- channels in complex with pore-blocking toxins had been solved [17, 18]. However, the lack of experimental data can be compensated through the use of molecular modeling tools.

Peptide blockers may have strong affinity (dissociation constants in the pico- and nanomolar range) for several closely related members of the K_V_-channel family. To this end, the pharmacological potential of toxins is exploited to increase selectivity via structure optimization. One such example is ShK-186, a synthetic analog of the sea anemone peptide, which blocks the potential-gated K_V_1.3 channel in the picomolar range. It is currently under investigation in phase 1B clinical trials as a therapeutic for autoimmune diseases [19]. Another feature of selective toxins is their potential use for ion channel discovery, ion channel distribution, and identification of a role for a channel in pathologies. Major efforts to address these issues are based on molecular modeling.

We will begin this review by summarizing published research on the molecular modeling of the interactions between K_V_-channels and pore blockers, including docking and binding energy calculations. A general overview of theory and approaches in modeling of ion channels, as well as a summary of the literature on the use of molecular modeling of ion channels outside the family of K_V_-channels, is beyond the scope of this review, and the reader is referred to the recent comprehensive publication by Gordon *et al *[20].

## THE STRUCTURE OF KV-CHANNELS AND PEPTIDE PORE BLOCKERS IN FREE AND COMPLEX FORMS

X-ray crystallography, nuclear magnetic resonance spectroscopy (NMR), and electron microscopy offer unique advantages for determining the molecular architecture of potassium channels bound to toxins. To date, numerous structures of peptide pore blockers, 12 potassium channels in free form [21] and two in complex with charybdotoxin have been solved and reported. Analysis of the atomic structure of K^+^ channels in bound and free states has been hampered by technical difficulties with extraction, purification, and crystallization of membrane proteins. These challenges add value to each resolved structure that provides structural details of the activation mechanisms, function, and interaction-induced changes [22].

## STRUTURAL CHARACTERIZATION OF POREBLOCKING PEPTIDES THAT BLOCK K+ CHANNELS

Most potassium channel blockers found in scorpion venoms belong to the α-KTx family. The sea anemone toxin ShK and dendrotoxins from mamba snake venom (α-DTX, DTX-I, DTX-K, δ-DTX) are also among the KV-channels blockers. Certain peptides can block both the K_V_- and K_Ca_-channels (*Table 1*). NMR measurements and, to some extent, X-ray analysis have helped understand the structures of many pore blockers (*Table 1*), laying the groundwork for subsequent interface analysis.

**Table 1:** Important peptide blockers of K_V_-channels with experimentally determined structures

Name	Abbreviation	Subfamily	Pdb code (reference)	Target channels
Charybdotoxin	ChTx	α -KTx 1.1	2CRD [23]	Kv1.1, 1.2, 1.3, 1.6 K_Ca_1.1, 3.1
	Lq2	α -KTx 1.2	1LIR [24]	K_V_, K_Ca_, K_IR_
Noxiustoxin	NTX	α -KTx 2.1	1SXM [25]	K_V_1.2, 1.3
Margatoxin	MgTx	α -KTx 2.2	1MTX [26]	K_V_1.1, 1.2, 1.3
Chongotoxin	HgTx	α -KTx 2.5	1HLY [27]	K_V_1.2, 1.3
Kaliotoxin	KTx	α -KTx 3.1	1XSW [28]	K_V_1.1, 1.2, 1.3, 1.6 K_Ca_3.1
Agitoxin	AgTx2	α -KTx 3.2	1AGT [29]	K_V_1.1, 1.2, 1.3, 1.6
	BmKTx	α -KTx 3.6	1BKT [30]	KV1.3
	OSK1	α -KTx 3.7	1SCO [31]	K_V_1.1, 1.2, 1.3, K_Ca_3.1
	Pi1	α -KTx 6.1	1WZ5 [32]	K_V_1.2
Maurotoxin	MTX	α -KTx 6.2	1TXM [33]	K_V_1.1, 1.2, 1.3, K_Ca_3.1
	HsTx1	α -KTx 6.3	1QUZ [34]	K_V_1.1, 1.3, K_Ca_3.1
	Pi4	α -KTx 6.4	1N8M [35]	K_V_1.2
	BmP01	α -KTx 7.2	1WM7 [36]	K_V_1.3
Cobatoxin	CoTx1	α -KTx 10.1	1PJV [37]	K_V_1.2
	Vm24	α -KTx 23.1	2K9O [38]	K_V_1.3
	ShK		1ROO [39]	K_V_1.1, 1.3, 1.6, 3.2, K_Ca_3.1
Dendrotoxin-K	DTX-K		1DTK [40]	K_V_1.1

**Fig. 1:**
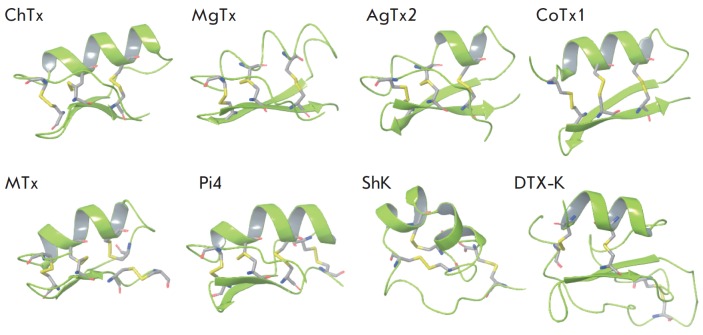
Structures of charybdotoxin (ChTx), margatoxin (MgTx), agitoxin-2 (AgTx2), cobatoxine-1 (CoTx1), maurotoxin (MTx), toxin Pi4, ShK toxin, and dendrotoxin DTX-K in a ribbon representation. The cysteine residues that form disulfide bridges are shown in rods.

**Fig. 2:**
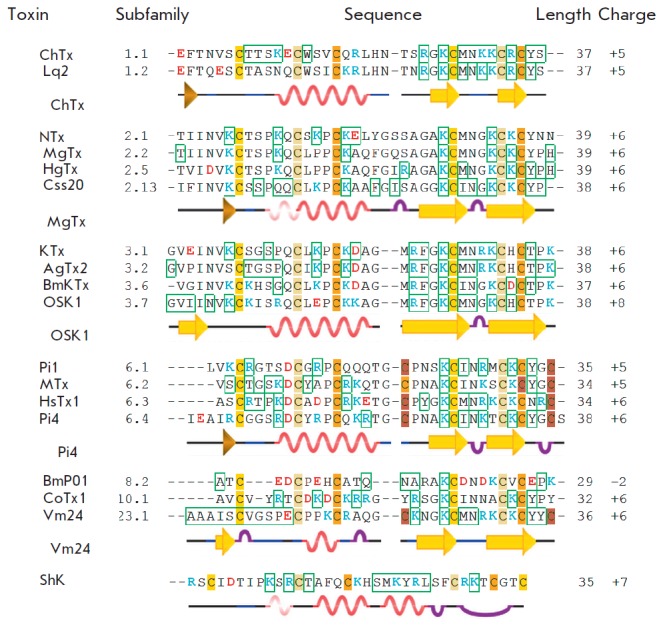
Alignment of the amino acid sequences of toxins blocking K_V_-channels. Residues that form contacts with a variety of channels according to the simulation are marked with green boxes. Cys residues are highlighted in yellow and shades of brown (residues forming disulfide bridges are shown with the same color), positively and negatively charged residues are in blue and red, respectively. The secondary structure of several toxins is shown (according to PDB identifiers, Table 1). Beta-strands are represented in yellow arrows, alpha-helices in bright wavy lines, 3/10-helices in pale wavy lines, turns in purple arcs, and curves in blue lines. Unstructured areas are shown in black lines

Alpha-KTx peptides (more than 50 NMR structures available) adopt a common alpha/beta scaffold (*Fig. 1*, *Fig. 2*), comprising an α-helix and two or three β-sheets. The structural differences lie in the sequence length (ranging from 29 to 40 residues) and amino acid composition (*Fig. 2*) affecting the alpha helical and beta structural regions.

Alpha-KTx peptides contain three or four disulfide bonds which hold the conformation in a rigid state. The number of disulfide bonds is fixed within each subfamily, except for the α-KTx6 subfamily, whereby disulfide bond formation is favored between cysteine residues 1 and 5, 2 and 6, 3 and 7, 4 and 8, as is in Pi4 toxin (*Fig. 1*, *Fig. 2*). By contrast, maurotoxin (*Fig. 1*, *Fig. 2*) and spinotoxin (α-KTx6.13) contain disulfide bonds between cysteine residues at positions 1 and 5, 2 and 6, 3 and 4, 7 and 8. Structural studies suggest more than one disulfide bonding pattern without changing the overall conformation in light of the fact that sulphur atoms in cysteines at 3, 4, 7 and 8 are in close proximity to one another [41].

At neutral pH, α-KTx toxins carry an overall positive charge that varies between +2 to +8, whereas negatively charged residues are more clustered in the N-terminal half of the peptide (*Fig. 2*). An exception is α-KTx8 toxins, in particular, BmP01 [41] with a net charge of –2 (*Table 1*, *Fig. 2*), a peptide with activity toward Kv1.3 channels [42].

ShK and DTX-K dendrotoxin peptides are closely related in terms of sequence length and disulfide bonding pattern to scorpion venom toxins but differ in structure (*Fig. 1*, *Fig. 2*).

## EXPERIMENTALLY SOLVED STRUCTURES OF POTASSIUM CHANNELS

**Fig. 3:**
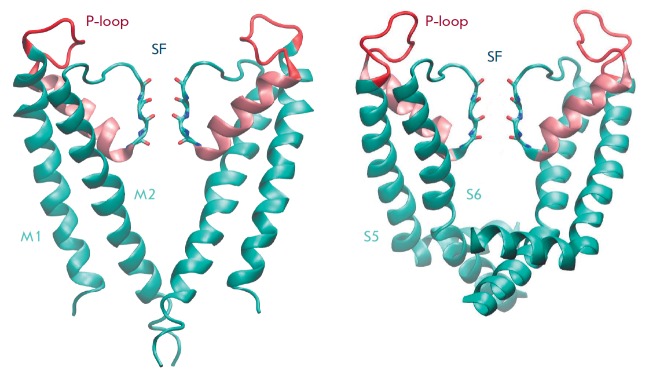
Crystallographic structure of the KcsA channel (pdb code 1BL8) (left) and the pore domain of the KV1.2 channel (pdb code 2A79) (right) in a ribbon representation. For clarity, only two α-subunits out of four are shown. The P-loops are shown in red; P-helices, in pink. Backbone oxygen atoms of the residues forming the SF are shown. Potassium ions are not shown

**Fig. 4:**
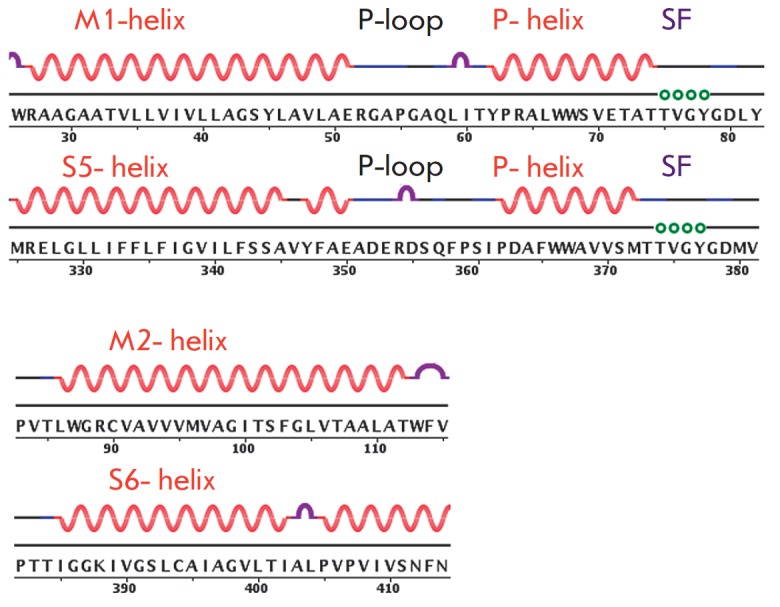
Comparison of the primary and secondary structures of the KcsA channel (pdb code 1BL8) and the pore domain of the K_V_1.2 channel (pdb code 2A79). Circles indicate residues that are in contact with the K^+^ ions in the SF (a fragment of the motif TVGYG). The secondary structure is presented as in Fig. 2.

The KcsA prokaryotic potassium ion channel was among the first to be structurally resolved by X-ray crystallography (*Table 2*). The pore forms a cone-like structure composed of four α-subunits. Each subunit is made up of two transmembrane α-helices M1 and M2 and the pore region P, which can be divided into a P-loop, a P-helix, and a selectivity filter (SF, *Fig. 3*). SF is formed by oxygen atoms of amino acid residues comprising a TVGYG motif (in some ion channels TIGYG or SVGFG) characteristic of potassium channels. The overall length is 12 A, and the cavity can only accommodate K+ ions. Although KcsA is not voltage-gated, the TVGYG motif in the filter region seems to be conserved in eukaryotic K_V_-channels (*Fig. 4*).

**Table 2:** Structures of K_V_-channels alone and in complex with charybdotoxin used in homology modeling studies

Description	pdb-code (resolution, Å) (reference)	Reporting studies
KcsA channel	1BL8 (3.20 Å) [54]	[55]
K_v_AP channel	1ORQ (3.20 Å) [56]	[57]
K_v_1.2 channel	2A79 (2.90 Å) [43]	[58, 59]
	3LUT (2.90 Å) [44]	[60-62]
Chimaeric KV1.2-2.1 channel	2R9R (2.40 Å) [45]	[63-68]
Mutant KcsA in complex with ChTx	2A9H^#^ [17]	[69, 70]
K_v_1.2-2.1 in complex with ChTx	4JTA (2.50 Å) [18]	[71]

^#^The structure was solved by NMR in contrast to the other structures solved by X-ray crystallography.

A total of 60 possible states of KcsA have been identified, which include open or closed conformation, in complex with low-molecular-weight ligands, with ions at the ion binding sites in the selectivity filter (K^+^, Cs^+^, Rb^+^, Tl^+^), including mutation-induced conformations that mimic the structural features of the pore regions of eukaryotic potassium channels. The atom coordinates of the P-loop and the docking position of pore blockers remain unaltered across all conformational states.

Another prokaryotic potassium channel with a refined X-ray structure is the archeal voltage-gated K_V_AP channel (*Table 2*). The archeal channel differs from KcsA in a more complex structure of α-subunits containing six helices (S1–S6). The helices S5 and S6 of four α-subunits, like the M1 and M2 helices of the KcsA channel, are arranged to form a cone-shaped structure with a pore, while S1–S4 helices make up a voltage-sensor domain (VSD-domain).

Only a few eukaryotic potassium channels have so far been described (*Table 2*). Among the first was the K_V_1.2 channel [43], later refined to a higher atomic resolution [44]. As is the case with KVAP, the α-subunits of K_V_1.2 are each composed of six helices (S1–S6) lining the central pore (S5–S6) and the VSD-domain (S1–S4). The K_V_1.2 refined structure clearly shows the spatial arrangement of the loops S1–S2, S2–S3 and S3–S4 which connect the helices of the VSD-domain and side chains of residues in the helices S2, S4, and the loop S5–P. A crystallographic analysis of K_V_1.2 revealed structural homology to the pore domains of both K_V_AP and KcsA (*Fig. 4*). Despite a shared homology of 65%, the structure and adjacent sequence of the selectivity filter demonstrate a high conservation (the root mean-square deviations of the Cα carbon atoms positions for residues 65–85 in KcsA from their positions in the K_V_1.2 are within 0.8 A). However, the pore domains of K_V_1.2 and KcsA slightly differ in length and conformation of the P-loops and cytoplasmic side of the transmembrane helices (*Fig. 3*). A comparative crystallographic analysis of the K_V_1.2 channel showed that the P-loop conformation seems to be flexible: the most common conformation found in structures with PDB IDs 2A79 and 3LNM and a unique conformation assigned under PDB ID 3LUT.

Although the pore domains of K_V_1.2 and K_V_AP are structurally related, the spatial organization of VSD-domains exhibits dramatic variations. It is likely that these differences are brought about by changes in the structure during extraction and crystallization of K_V_AP [43].

Yet, the structures of other eukaryotic K_V_-channels remain to be determined, but based on the high level of homology the structure of their pore domains is anticipated to be similar to that of K_V_1.2.

## RESOLVED STRUCTURES OF PATASSIUM CHANNELS IN COMPLEX WITH PEPTIDE BLOCKERS

The first crystal structure of an ion potassium channel bound to a pore-blocking peptide was solved by NMR for a surrogate KcsA ion channel and charybdotoxin (*Table 2*) utilizing structural knowledge of pore channels and blockers [17]. The wild-type KcsA, insensitive to eukaryotic potassium channel blockers, was modified by mutating three residues (Q58A, T61S, R64D). These mutations enhanced structural similarity to eukaryotic Shaker K_V_ channels and increased affinity for charybdotoxin. Another three mutations (F103Y, T107F, L110V) were introduced to the central region to increase homology to human K_V_11.1. A NMR spectral analysis showed that charybdotoxin binding induces conformational changes in the channel structure, whereas the M1 and M2 helices remain unperturbed. Following binding, the toxin backbone remains rigid. There is evidence defining a blocking mechanism for K27 charybdotoxin at the toxin-pore interface.

**Fig. 5:**
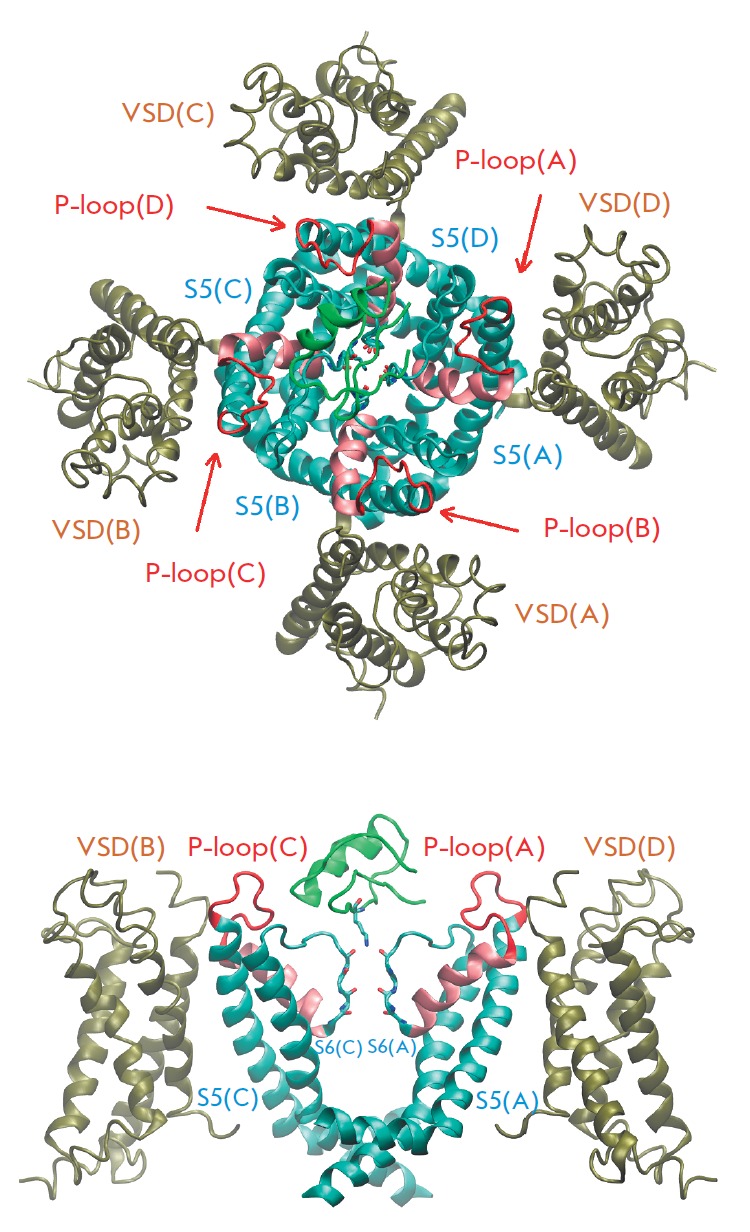
Crystallographic structure of charybdotoxin in complex with the chimaeric K_V_1.2-2.1 channel (PDB code 4JTA) is shown in a ribbon representation (top view and side view). For clarity, only two α-subunits are shown. The voltage-sensitive domain (VSD) is shown in olive green. The toxin is shown in bright green, and its residue K27 projecting into the pore is shown in a rod representation. The other designations are the same as in Fig. 3. The letters A, B, C, and D denote the subunits of the channel.

Recently, Banerjee *et al *reported on the structure of a chimaeric K_V_1.2–2.1 channel in complex with charybdotoxin resolved by X-ray crystallography (*Table 2* ,*Fig. 5*). The voltage-sensor paddle (the S3b–S4 loop segment) of K_V_1.2 was replaced by the voltage-sensor paddle of KV_2_.1 as described previously [45]. The crys tal structure demonstrated that the chimaeric channel does not undergo conformational changes upon toxin binding [18].

Studies of the structures of pore-blocking toxins such as charybdotoxin in complex with Kv channels (mutated KcsA and chimaeric K_V_1.2–2.1) reveal that the binding involves most of the interactions between amino acid residues of the toxin molecule and the channel loops. Importantly, the P-loops exhibit the widest variation across domains in K_V_-channels and it is highly likely that it is this variation that accounts for the varying degrees of toxin affinity to the target channels.

## HOMOLOGY MODELING OF POTASSIUM CHANNELS AND THEIR COMPLEXES

In the case when only a few potassium channels have atomic resolution models in free form and in complex with pore blockers, homology modeling happens to contribute to the construction of a structure of target K_V_-channels and their complexes. This approach [46] relies on modeling a three-dimensional structure of the target protein using an experimentally determined structure of a related protein (the template). The steps in homology modeling are the following [47]:

Template identification: search for homologous proteins with elucidated structures, for example, using BLAST [48] or FASTA [49];

Target-template amino acid sequence alignment, for example, using CLUSTALW [50]; and

Model generation by threading the sequence on the template sequence, replacing residues and building missing parts, for example, using the Modeller software [51].

Fortunately, the Swiss-model online server [52] can run all three steps in automated mode.

An important requirement for model generation is that the sequences should have more than 20–30% sequence identity [53]. The pore domains of potassium channels readily meet this criterion: the sequence identity of these domains of KV-channels share over 80%, with most of the variability located in the small pore loop. The atomic resolution of K_V_1.2–2.1 bound to charybdotoxin [18] is in good agreement with other experimental findings, which indicates that the VSDdomain is not involved in the binding. For this reason, modeling of complex structures of pore blockers with* K*_V_-channels is only focused on pore domains.

The sequence homology of the pore domains of K_V_- channels and the prokaryotic KcsA channel is approximately 30%, thus making KcsA, together with other structures, an optimal template for modeling studies [55, 69, 70, 72] (Table 2). This is justified by experimental measurements demonstrating that chimaeric KcsAKv1. x channels (KcsA, in which the P-loop is replaced by the P-loop of Kv1.x, x = 1, 2, 3, 6) retain affinity, characteristic of Kv1.x channels [73-75].

Homology modeling is used in conjunction with Brownian dynamics (BD), molecular docking, and molecular dynamics (MD) simulations.

## BROWNIAN DYNAMICS

A Brownian dynamics is a case of stochastic dynamics treating the movement of molecules as solid bodies under the influence of external perturbations and friction forces which simulate the interaction with solvent molecules and random forces. BD has been widely used for simulating ion transport [76, 77] and, to some extent, binding of toxins to ion channels [78, 79]. It is assumed that channels and blockers have rigid structures, the solvent is implicit, and the cell membrane is represented as an idealized dielectric slab [20]. Brownian dynamics simulations with these key assumptions allow one to decrease computational costs and speed up the overall process for macroscopic effects to be detected [80].

Using Brownian dynamics simulations, the predicted structure of Lq2 toxin (*Table 1*) bound to mutated KcsA revealed the key amino acids of the toxin responsible for binding [78]. Since this model assumes that the blocker and channels have rigid structures, the conformational flexibility of the blocker was simulated based on all 22 structure variants available for Lq2 in the PDB-file. One variant yielded a model consistent with experimental observations. The channel flexibility was excluded from the analysis.

Structural insights into conformational flexibility in Brownian dynamics simulations could be gained by incorporating MD to the protein structures, as was successfully applied to K_V_1.3 in complex with agitotoxin, charybdotoxin, kaliotoxin, margatotoxin, and noxiustoxin [79]. Electrostatic energy calculations in complexes have been shown to tightly correlate with logarithms of dissociation constants (R^2^ = 0.60). However, this was not true for kaliotoxin, which can be explained by an inappropriate structure (PBD ID 1KTX), showing poor structural alignment with agitotoxin and OSK1. Refined kaliotoxin structures (PBD ID 1XSW, 3ODV) do not have this drawback.

## MOLECULAR DOCKING

Molecular docking is a computational technique that predicts the preferred orientation and conformation of a ligand that binds to a target binding site. Toxins in complex with potassium channels have been widely explored in molecular docking studies [38, 62, 80].

In a protein-protein docking algorithm, one molecule is fixed in space and the other is translated and rotated around, exploring possible orientations and conformations. The quality of each possible fit is calculated by a scoring function that takes into consideration complementarity, electrostatic interactions, van der Waals repulsion, desolvation energy, internal energy strain (deformation of valence bonds), hydrogen bonding, and aromatic group interactions.

Molecular docking is carried out using a suite of docking tools, such as AutoDock (http://autodock. scripps.edu/) [81], RosettaDock [82], BiGGER [83], HADDOCK (http://haddock.science.uu.nl/) [84], or ZDOCK (http://zdock.umassmed.edu/) [85]. The latter three software are the most common for channel blocker complexes simulations [37, 86, 87].

A distinctive feature of HADDOCK is that it performs docking of large and flexible peptide ligands. This software was used to develop a comparative docking protocol predicting the selectivity profiles of α-KTx toxins [87]. Channel-toxins interactions were successfully modeled using this protocol. However, the HADDOCK scoring function failed to accurately predict affinity scores, which impedes correct ranking.

Molecular docking can be employed to investigate the conformational flexibility of toxins. Corzo *et al *attempted* in silico *modeling of the interactions of Css20, a novel voltage-dependent K^+^-channel blocker, with potassium channels [58]. A total of over 1,000,000 structures were generated, of which the 2,000 best ranking complexes were visually inspected. Unfortunately, this approach is computationally laborious and has insufficient scoring functions, which generate multiple top scoring hits, among which the best scoring pose is ranked inaccurately. A possible solution to this problem is small-scale screening, followed by molecular dynamics simulations and determination of binding constants through free energy calculations [20].

## MOLECULAR DYNAMICS

In molecular dynamics simulations, the temporal evolution of interacting atoms or particles is determined by numerically solving the equations of motion. The accuracy of MD algorithms depends on force fields: i.e., potential energy functions and parameters deduced from experimental data and quantum-mechanical measurements. Current generation force fields provide energy estimates with remarkable accuracy [88]. Full-atomic force fields such as OPLS-AA and CHARMM variants take into account all atoms in the system. Alternatively, force fields with united atoms like GROMOS and OPLS-UA treat heavy nonpolar atoms (carbon, sulphur) and related hydrogen atoms as single interaction entities [89].

The NAMD software package [90], together with CHARMM [91], including variants such as CHARMM22, CHARMM27 and CHARMM36, has been the most commonly used for K_V_-channel modeling (Table 3). The CHARMM27 force field is optimized for DNA, RNA and lipids, and in combination with CHARMM22, for DNA-protein interactions. Although CHARMM27 seems to be not intended to simulate protein-protein interactions, the free energy binding estimates generated by this software are in good agreement with experimental observations [60, 66, 92], (*Table 3*). Unlike CHARMM22, CHARMM36 has been extended to incorporate optimized parameters for the conformational space upon protein folding, protein as sembly into complexes, and the relevant conformational changes [93].

**Table 3:** Cardiotoxins: properties and conformational characteristics

Channel (pdb-code of template)	Toxin (pdb-code of structure or template)	MD force field	ΔG_calc_, kcal/mol	ΔG_exp_, kcal/mol	reference
K_V_1.1 (2R9R)	HsTx1 (1QUZ)	NAMD, CHARMM36	-10.1 ± 0.6	-11.1 ± 0.1	[68]
	ShK (1ROO)	NAMD, CHARMM22	-14.3 ± 1.1	-14.7 ± 0.1	[63]
	ShK-K-amide (1ROO)	NAMD, CHARMM22	-11.8 ± 1	-12.3 ± 0.1	[64]
	ShK-K18A (1ROO)	NAMD, CHARMM27	-11.7 ± 0.7	-11.3 ± 0.1	[66]
K_V_1.2 (2R9R)	HsTx1 (1QUZ)	NAMD, CHARMM36	-8.9 ± 0.6	-9.5 ± 0.1	[68]
	ShK (1ROO)	NAMD, CHARMM22	-10.1 ± 1.1	-11 ± 0.1	[63]
	MTx (1TXM)	NAMD, CHARMM36	-12.6	-12.6	[92]
K_V_1.3 (3LUT)	ChTx (2A9H)	NAMD, CHARMM27	-10.4	-12.5	[60]
	MgTx (1MTX)	NAMD, CHARMM36	-11.5	-13.9	[62]
K_V_1.3 (2A79)	ChTx (2CRD)	GROMACS, OPLS-AA	-26 ± 1	-11.4 ± 0.2	[59]
K_V_1.3 (2R9R)	HsTx1 (1QUZ)	NAMD, CHARMM36	-14.0 ± 0.6	-14.9 ± 0.2	[68]

Current MD simulations allow one to investigate toxin- channel complex interactions with the lipid membranes which affect the predicted structure of a toxin bound to the VSD-domain of a channel [94]. The lipid bilayer *per se *is not relevant for modeling complexes of pore-blocking peptides [55]. This is in good agreement with experimental data and calculations of complex formation free energies optimized by a MD analysis [95, 96]. Discarding the lipid-protein interactions helps reduce the computational burden and allows one to extend MD trajectories and the computational space. Another approach to addressing computational challenges is the use of an implicit solvent model, in particular, a generalized Born solvation model. Regardless of the solvation model utilized, MD simulations are carried out in the range of 15–20 [68, 92] to 40–50 ns trajectories [62, 97].

In certain cases, complex formation is examined using steered MD. These simulations are based on the pulling of a number of toxin residues to the corresponding residues of the channel, provided that these pairs of residues are key determinants in the complexation. The advantage of this method is that it considers experimental data on the interacting residues, the contacts of which are assumed more accurate. Steered MD has been well used in studies on a K_V_1.2 channel complex with maurotoxin [92] and a K_V_1.3 channel complex with margatoxin and chongotoxin [62].

## ENERGY CALCULATIONS OF CHANNEL-TOXIN COMPLEXES

The quality of predicted protein structures is validated by the free-energy change ΔG. These calculations are of paramount importance for determining the energetic favorability of a given biochemical reaction, as well as ligand binding to receptors and downstream conformational changes [98].

Binding free energies of ligand binding to potassium channels are determined by the potential of mean force calculations using an umbrella sampling scheme for conformational states [99]. This technique predicts ΔG values that are consistent with experimental data (*Table 3*), thus suggesting the high accuracy of predictions made by a numeric model and utility for constructing peptide blockers with the desired properties. Khabiri *et al *[59] obtained ΔG values which were at least two-fold lower than the calculated ones due to the differences in the ionic strength in computational and experimental conditions. Alternatively, the discrepancy may be explained by the lower accuracy of the OPLS-AA force field versus CHARMM variants (Table 3).

Another, but less commonly used, approach to free energy estimation is MM-PBSA (molecular mechanics/ Poisson–Boltzmann surface area), which combines the molecular mechanics potential energy in vacuum and the Poisson–Boltzmann surface area method for calculating free energies of solvation. The MM-PBSA method is incorporated in AMBER [100] and GROMACS [101] and is qualitatively consistent with experimental measurements in the case of free energies of toxin binding to channels [102].

## THE USAGE OF MOLECULAR MODELING TECHNIQUES

Modeling of the structures of KV-channels in complex with naturally occurring and chemically designed peptide blockers provides valuable insights into structural and interactional properties, key atoms between the two docked structures, and could aid in understanding the observed potency and range of activities for pore blockers of interest.

Experimental findings on the binding of intact and mutant Pi1 to K_V_1.2 were explained when these complexes were homology modeled using maurotoxin (*Table 1*) and KcsA template structures [86]. After a series of docking calculations, the peptide residues R5, R12, R28, and K31 were found to contribute in stabilizing the complex.

A similar approach was followed in conducting a structural characterization of the interface of K_V_1.2 in complex with cobatoxin (*Table 1*) and its synthetic analog ACoTX1, which contains T7P and D9Q substitutions [37]. The inspection revealed that the orientation of the dipole moment does not much contribute to its selectivity, leading to a conclusion that molecular modeling is particularly important in screening for toxin analogs with high affinity and/or selectivity.

Notably, docking experiments clarified the remarkable selectivity of Vm24 and its analogs towards K_V_1.3 [38]. These selectivity factors were assigned to tight contacts between the N-terminal portion of Vm24 and the channel molecule, which is not the case for other α-KTx family toxins (*Fig. 2*).

Jin *et al *[55] explored the space of possible poses of ShK in complex with KV1.3 by combining molecular docking and molecular dynamics methods and discovered two favorable interaction states. In the first pose, the channel selectivity filter was occluded with the K22 residue; in the other, with the R24 residue. MM-PBSA calculations of ΔΔG upon substitutions to Ala changes in the peptide molecule, together with empirical data on complex dissociation constants, favored the second model with the R24 residue.

The mutant cycle analysis has emerged as a valuable tool for the study of toxin-channel complexes. This approach was first applied to characterize Shaker channel binding sites relative to the agitoxin 2 structure [103]. It is logically clear that substituting residues in the structures of a toxin and a channel will affect the dissociation constant if the targeted residues are key to binding that can be defined by the coupling coefficient. Knowledge of the details of the key contacts between the toxin and channel allows one to model complex structures in docking simulations. Owing to algorithm advances in free energy calculations, mutant cycle analysis has become a well established approach commonly used in docking studies [55, 57, 95, 102, 104].

Yi *et al *[105] reported on a specific binding of maurotoxin to the K_V_1.2 channel explored by molecular dynamics and molecular docking screening. Calculation of the binding free energy by MM-PBSA upon substitutions of Ala residues based on experimental findings identified the most plausible candidate structure with the key residues located at positions K23, I25, and Y32 in the toxin and R254, F359, P360, D379, V381, and T383 in the channel.

Molecular modeling of the spatial structures of ADWX-1 in complex with K_V_1.1 and K_V_1.3 [106] revealed three favorable pore loop conformations in complex with pore blockers: open, half-open/halfclosed, and closed. In the open conformation of pore loops, the peptide interacts with the residues outside the selectivity filter and the pore loop has no effect on the toxin channel interface. This is how, in accord with reference [105], the maurotoxin peptide seems to bind to the K_V_1.2 channel. When the pore loop conformation is half-open/half-closed, the loops play a minor role in stabilizing the interaction, as previously shown for ADWX-1 in complex with K_V_1.1 and K_V_1.3. In the closed structure, the binding interface is formed by both the pore loops and the region around the selectivity filter, driving affinity and selectivity for toxin binding.

It has been recently shown [60] that the contribution of electrostatic interactions dominates over the contribution of van der Waals forces in complexes of charybdotoxin, OSK1, and ShK with K_V_1.3. The critical amino acid residues dictating the toxin orientation were lysine and arginine: K27 and R25 in charybdotoxin, K27 and R24 in OSK1, K22, and R11 in ShK (*Fig. 2*). Binding energy values calculated by mean force potentials were found to be in agreement with published experimental studies (*Table 3*). Unfortunately, providing high accuracy imposes a computational burden, which limits the application of numerical modeling of binding energies and highlights the need for improved computational efficiency. Of note, the findings reported by Jin *et al* and Chen *et al *on ShK in complex with K_V_1.3 are not consistent [55, 62] and require further clarification.

MD screening for toxin-induced conformational changes in the chimaeric KcsA-K_V_1.3 channel in complex with kaliotoxin [72] led to a model in which the Y78 residue of the toxin and D80 residue of the channel changed positions following interface formation. The simulation was validated using NMR measurements.

The nascent research efforts to study potassium channel blockers led to an idea [107], which matured and expanded afterwards, that the ability of toxins from different species to target K_V_-channels is attributed to the presence of the functional dyad: a lysine residue that recognizes the selectivity filter and a hydrophobic residue (Tyr, Phe or Leu) 6–7 A away from SF. With advances in computational techniques, including molecular modeling, the specificity for K_V_- channel binding was shown to depend on other aminoacid residues. The extent to which the functional dyad contributes to the binding energy also varies depending on toxin fold and other amino acid resides [108]. Molecular modeling data clearly demonstrate that the toxin-channel interface is caused by contacts between multiple residues. This network of binding interactions differs not only among groups and subfamilies, but even among highly homologous peptides.

Improved selectivity of K_V_-channel blockers has been the focus of pharmacological studies, because naturally occurring toxins have the peculiarity of possessing affinity for a range of channel types. The identification of the differences between interacting resides of the toxin and channel, as determined by molecular modeling, offers much opportunity for site-directed mutagenesis to modulate toxin binding to a given channel.

The Css20 toxin acts on K_V_1.2 and K_V_1.3 versus K_V_1.1 and K_V_1.4 [58]. Molecular modeling showed that the key amino acid residues of K_V_1.2 and K_V_1.3, which are in contact with Css20, are located around the selectivity filter and in the P-loop (7 out of 8 residues differ among the channels). The K28 residue was found to be crucial for binding K_V_1.2 and K_V_1.3, and the Q11, I30, K33, and Y37 residues form favorable contacts with only K_V_1.2. In addition, new contacts may arise with KV1.2 upon substitution of A19 and A20 for the positively charged Arg or Lys. It is suggested that tailored mutations can enhance the selectivity of Css20 analogs for K_V_1.2 and K_V_1.3.

The same approach based on MD and the potentials of the mean force was applied to the bound complex OSK1 and K_V_1.1– K_V_1.3 channels in the search for amino acid substitutions increasing the activity of the peptide [65]. The authors revealed that K9S and S11R could lead to enhanced potency in blocking K_V_1.3 with decreased activity toward K_V_1.1 and K_V_1.2. The mutant OSK was 10,000-fold more specific for K_V_1.3 than for K_V_1.1 and K_V_1.2. The potency of OSK1 for Kv1.3 was increased by 100-fold.

In a site-directed mutagenesis study, Han *et al *[102] introduced the G11R, I28T, and D33H substitutions into the BmKTX peptide to obtain a highly potent K_V_1.3-blocking peptide, named ADWX-1 [102]. The functional residues of ADWX-1 in complex with K_V_1.3 were identified using a structural model of the ADWX- 1-K_V_1.3 complex constructed by molecular modeling. Energy binding estimates for ADWX-1 and its variants (R23A, F24A, K26A, N29A, T35A) established an important role for positively charged residues in recognizing K_V_1.3. In addition, the R23A and F24A substitutions provide steric hindrance to the contact of the key K26 residue with the channel pore. The experimentally determined affinity of ADWX-1 for K_V_1.3 was increased by 100-fold relative to the native BmKTX peptide. The selectivity of ADWX-1 toward K_V_1.3 was increased by 340-fold and > 105 -fold versus K_V_1.1 and K_V_1.2.

## CONCLUSION

Over 30 wide-scale studies utilizing computational simulations have been carried out to provide insights into the structure of potassium channels alone and in complex with toxins. *In silico *approaches contributed to the elucidation of toxin-channel interactions, revealed important molecular clues on the mechanisms of selectivity and affinity of toxins, and laid the basis for a rational design of pore-blocking peptides with tailored properties.

The most commonly used modeling approach to resolving toxin-channel structures involves homology modeling, molecular docking, and molecular dynamics techniques, which could be combined with MM-PBSA free energy computations or the potentials of the mean force. Each algorithm is executed in a defined computational context of parameters depending on an investigator’s preferences and experimental design. Currently, there is a clear trend away from Brownian dynamics and implicit solvation approaches to simulations in explicit water. MM-PBSA free energy computations are discarded in favor of the potentials of the mean force.

Further improvement in molecular modeling algorithms requires the availability of high-resolution structures of channels from major families and/or toxin-channel complexes. However, the accuracy of the modeling depends on force fields, high throughput docking algorithms, and free-energy calculations to determine the binding free energies of toxins.

Due to their biological and medical significance, potassium channels offer great promise to encourage novel computational approaches that will minimize the computational burden and cost.

## Abbreviations

BD: Brownian dynamics
MD: molecular dynamics
PMF: the potential of mean force
X-ray: x-ray analysis
RMSD: root mean square deviation
SF: selectivity filter
NMR: nuclear magnetic resonance
αKTx: a family of channel blocker toxins from scorpion venom
KVchannels: voltage-gated potassium channels
MM-PBSA: molecular mechanics/Poisson-Boltzmann surface area
VSD: voltage-sensor domain
